# A Case Report: The Vicious Cycle of Schizophrenia, Substance Abuse, and Human Trafficking

**DOI:** 10.7759/cureus.76211

**Published:** 2024-12-22

**Authors:** Linda Gavric, Shani M Abraham, Thomas J Valente

**Affiliations:** 1 Internal Medicine, Lake Erie College of Osteopathic Medicine, Bradenton, USA; 2 Psychiatry and Behavioral Sciences, Lake Erie College of Osteopathic Medicine, Bradenton, USA; 3 Psychiatry, LifeStream Behavioral Center, Leesburg, USA

**Keywords:** antipsychotics, bipolar disorder (bd), human trafficking, manic episodes, marijuana use, pseudocyesis, schizoaffective, schizophrenia, stockholm syndrome, substance abuse

## Abstract

Patients with schizophrenia often find themselves in vulnerable situations because their cognitive impairments can make them more susceptible to exploitation and crime. A prevalent misconception is that schizophrenia is synonymous with violence, possibly fueled by selective media coverage that highlights instances of violent crimes involving individuals with schizophrenia. In reality, a large percentage of people with schizophrenia do not display violent behavior. In this case report, we will explore the circumstances of a 28-year-old female patient diagnosed with schizophrenia who was missing for four years, whose past medical history involves substance abuse, and who may also be a victim of human trafficking. We will examine these factors both separately and in conjunction, demonstrating how they all contribute to a vicious cycle of detrimental progress in mental health. We aim to challenge the myth that patients with schizophrenia are inherently violent and illustrate how they may be vulnerable victims in scenarios such as human trafficking.

## Introduction

Schizophrenia is defined as a serious psychotic disorder characterized by positive and negative symptoms. Positive symptoms include delusions, hallucinations (visual and auditory), and disorganized speech and behaviors. Negative symptoms include reduced emotional expression, cognitive impairment, and decreased motivation. According to the Diagnostic and Statistical Manual of Mental Disorders, Fifth Edition (DSM-5), the diagnosis is determined when two or more characteristic symptoms are present for one month. These include delusions, hallucinations, disorganized behavior, and negative symptoms. These signs of disturbance must persist for six months to be diagnosed as schizophrenia [1]. Initial treatment includes monotherapy with an antipsychotic other than clozapine, or an oral antipsychotic followed by the long-acting injectable (LAI) form of the antipsychotic medication if tolerable and sufficiently efficacious. Currently, available data estimates the prevalence of schizophrenia in the US to be between 0.25-0.64% [2]. Half of the individuals with schizophrenia have co-occurring behavioral and/or mental health disorders including substance abuse, anxiety disorders like obsessive compulsive disorder (OCD), post-traumatic stress disorder (PTSD), generalized anxiety disorder (GAD), panic disorder, and depression [2]. It is vital to consider substance abuse as research has depicted that schizophrenic patients who are also substance abusers have higher rates of unemployment and homelessness and lower rates of cognitive functioning compared to those who are not [3]. Patients with schizophrenia are placed in vulnerable positions due to their decreased cognitive ability which can make them susceptible to perpetrators of crime. One of the most common myths about schizophrenia is that it is synonymous with violence. This is perhaps due to selective media coverage of schizophrenic patients engaging in violent crime. A majority of individuals suffering from schizophrenia do not exhibit violent behavior. Research findings propose that only about 10% to 15% of this community engages in such actions, indicating that approximately 85% to 90% remain non-violent [4]. These findings indicate the possibility that patients with schizophrenia are more frequently victims of violent crimes. Based on research, individuals with schizophrenia living in community settings are around 14 times more likely to become victims of violence rather than commit these violent acts [4]. Cognitive deficits that impair their perception and awareness of the environment can increase these patients’ susceptibility to victimization [5]. In addition, the prejudice and misconception regarding schizophrenia can result in social isolation, exacerbating their vulnerability to human trafficking [5]. Human trafficking involves the sourcing and movement of individuals through approaches of coercion and deception, to manipulate these individuals. Exploitation can occur through domestic slavery, labor, or coerced sex work [5]. Human trafficking, a global conflict, affects around 49.6 million individuals and around 12 million children [6]. The most frequent form of trafficking in the United States is sex trafficking [6]. Disappointingly, individuals with mental and medical diseases are more susceptible to this form of manipulation [6]. People with mental illness who have been trafficked face the consequence of potentially worsening mental illnesses. This can be due to the neurochemical and functional changes of the brain related to stress-induced neuroplasticity-associated changes [7]. These individuals are also engrossed in an environment linked with other adverse factors such as crime, drug exposure, and poverty, expanding the negative impact on the victim's health [7]. A vicious cycle exists upon which chronic stress impacts the structure of the brain and function rendering the growing brain more vulnerable to the ill effects of abuse [7]. This article will emphasize the general indicators that a person may be at risk of being a victim of human trafficking as well as the importance of early detection of the victims of sex trafficking so they can be identified, protected, and treated for mental health issues. In this case report, we will examine a 28-year-old female patient diagnosed with schizophrenia with a history of substance abuse, who could be a possible victim of human trafficking. We will discuss these factors independently and how they intertwine and lead to a progressive vicious cycle. We hope to introduce a perspective that will depict how schizophrenic patients may actually at times be victims in situations like human trafficking. 

## Case presentation

A 28-year-old, African-American female patient presented to the behavioral care center involuntarily under the Baker Act. The Baker Act is a Florida law that allows for involuntary or voluntary mental health services lasting 72 hours for those who experience acute mental health crises. The patient did not have any identification on her and the police department was not able to identify her. For the past few years, she had been living with a man who claimed he had never seen her identification. The man claimed he had picked her up off the streets a few years ago and was allowing her to stay with him at his home. The patient seemed to suffer from a psychiatric break. She claimed she was a Victoria's Secret model, a stripper, was earning hundreds of millions of dollars a year, and was in the military as a snipper with permission to kill. The patient claimed she was pregnant and asked for a male prostitute to be ordered to the behavioral center. As she was taken to the behavioral center, she asked if she was allowed to die there. She hesitantly completed the safety check and was unwilling to provide a urine sample. She was unable to confirm her previous residence or whereabouts, though she stated that she was from another state and identified her birth date. This information was used to track the patient as a missing person from another state. The full name of the patient was also determined with this information.

At the center, the patient was observed to have an elevated mood. Her speech was loud and tangential, and she was not oriented to time, situation, or place. She became more agitated and began knocking on the walls. She continuously asked to leave the care center, claiming she was pregnant and had children at home. Upon further evaluation, the patient seemed to have argued with the man she had been living with but was unwilling to provide any further information on the topic. 

A diagnosis of schizophrenia was determined after a psychiatrist evaluated the patient. She met all the criteria for this diagnosis as mentioned earlier in this case report. The patient was deemed to be incompetent and a court order was granted for the patient to remain in involuntary inpatient placement. She was started on Abilify (aripiprazole) 30 mg by mouth (PO) daily for two weeks. She was then given LAI Aristada (aripiprazole lauroxil) 1064 mg and Aristada Initio (aripiprazole lauroxil) 675 mg intramuscular (IM). Over time, oral Depakote (valproate) 500 mg was added to help reduce the episodes of psychosis. The center reached out to her parents and her mother was appointed her guardian advocate. We followed the patient to note her progress after the initiation of medication.

After the second week of medication, we were able to sit with the patient and engage in a discussion where she was more aware of her surroundings and her diagnosis of schizophrenia. She had a greater insight into her mental health. During the conversation, the patient exhibited neologism, flight of ideas, distractibility, talkativeness, lack of eye contact, tangential thoughts, and delusions. She spoke in the third person and was more willing to engage in a conversation when asked questions from a third-person perspective. 

Throughout her stay at the center, the patient maintained a belief that she was pregnant, despite multiple negative pregnancy tests. She was reluctant to discuss the person she had been living with. When asked about her previous residence, she mentioned she was not allowed to discuss “him” and mentioned the paraphernalia at the residence. Since the beginning of her stay at the center, the patient consistently commented on her body's appearance and hair. She constantly apologized for how she presented herself physically, stating that she had to lose weight and comb her hair. During the third week of evaluation, the patient stated that she was happy on her medications, however, she still exhibited symptoms of mania, talkativeness, lack of eye contact, tangential thoughts, flight of ideas, and distractibility. These episodes opened up the possibility of a diagnosis of schizoaffective disorder, which will be further clarified in the discussion of this case report. She recognized and commented that she had been “delusional” about thinking she was pregnant. She stated that the faculty at the center had helped provide her with evidence that she was not pregnant. She continued to comment and apologize about her body weight and hair, stating it was not properly presented. 

We were able to document the patient's previous medical and mental health records and were aware that this information played a vital role in helping us understand how the patient had progressed over the years with her schizophrenia. Regarding her medical history, the patient had a long history of prior hospitalizations for episodes of psychosis. Her first hospitalization was at age 18 for one month and she had been hospitalized eight more times over the next three years. Her most recent hospitalization before the present time was in July 2020 where she presented with reports of being contaminated with parasites, paranoia about others wanting to harm her, thought disorganization, pressured speech, hyper verbality, reduced sleep, and significant food and water restriction with a weight loss of 15-20 lbs. Her previous health records depicted a history of noncompliance with medication. At age 21, she had a history of standing for prolonged periods leading to venous stasis and cellulitis requiring hospitalization with antibiotics and concern for lower extremity deep vein thrombosis (DVT). Prolonged standing was a concern for worsening psychosis and catatonia. Substance use was also a likely contributing factor since the patient had a medical history of cannabis use since high school. Records also showed that she drank alcohol regularly since age 16. Regarding her past psychiatric diagnosis, the records showed borderline personality disorder, obsessive-compulsive disorder, and body dysmorphia. Her past treatment records stated that the patient was placed on Abilify (aripiprazole) 5 mg with a plan to transition to aripiprazole LAI, given her long-standing history of medication nonadherence. She was also started on Ativan (lorazepam) 2mg for suspected catatonia. 

Her past social history showed that the patient was adopted at two months of age and not much was known about her biological mother. As the patient grew up, she was described as well-functioning child, doing well in school, getting good grades, and being very social. By the end of middle school, she became more withdrawn, avoidant, and anxious. She did not attend classes and avoided social gatherings. Around the junior year of high school, due to her withdrawn and anxious demeanor, the patient stopped attending school in person and decided to continue schooling from home. During her senior year, she developed a lot of phobias, including the fear of germs. She was started cutting herself and there were some family troubles during that time. After analyzing her behavior and history, we understood that the patient may have been exhibiting prodromal signs of schizophrenia. At that time, the patient also began to exhibit catatonic behavior, auditory hallucinations, and delusions. In her hallucination, she believed dementors were telling her she was not loved. She also began to exhibit thought broadcasting, believing everyone could hear her thoughts. The patient also had a previous history of engaging in a violent act towards a family member for which she was hospitalized. She was released from the hospital after the court ordered her as competent and she was released with the agreement that she would follow through with an outpatient treatment plan. However, upon discharge, the family did not hear from her at all. She was designated as a missing person until the current incident where she was brought to the behavioral center after four years. Below is a chronology of the patient's medication history obtained through her limited medical records (Table 1).

**Table 1:** Timeline of patient’s prescription history LAI, long-acting injectable; IM, imtramuscular; PO, oral.

Date	Prescription
2020	Abilify (aripiprazole) 5 mg PO, Ativan (lorazepam) 2 mg for suspected catatonia
Mid-August 2024	Abilify (aripiprazole) 30 mg PO daily for 2 weeks
End of August 2024	Aristada LAI (aripiprazole lauroxil) 1064 mg every 2 months, Aristada Initio (aripiprazole lauroxil) 675 mg IM
September 2024	Depakote (valproate) 500 mg PO

## Discussion

Schizophrenia is one of the most common major mental diseases. Since it is a chronic illness, the prognosis involves multiple relapses and admissions, with a proportion of patients relying on long-term institutional care. The onset of schizophrenia in a majority of patients occurs in younger adults. Since genetics is a strong predisposing risk factor, the calculated risk of the offspring having schizophrenia when both parents also have schizophrenia is close to 37% [8]. When one parent has schizophrenia, the risk is closer to 14% [8]. The current hypothesis states that most cases of schizophrenia result from polygenic gene interaction and possible extrinsic factors including drug abuse and trauma. Studies have shown that specific candidate genes associated with schizophrenia include neuregulin, dysbindin, proline dehydrogenase, and catechol-O-methyltransferase [8]. Guanylate-binding protein 2 (GBP2) is another gene that was replicated and upregulated in five studies assessing gene expression in schizophrenia [9]. In our case, the patient was adopted, hence no conclusions could be made about her genetic predisposition to schizophrenia.

Our patient presented with symptoms that included delusions, hallucinations, disorganized speech/behavior, unusual thought processes, tangential thoughts, flight of ideas, lack of eye contact, distractibility, talkativeness, and neologism. As defined in the introduction, schizophrenia is a chronic mental illness that affects daily functioning. The symptoms can present as positive (hallucinations, delusions, disorganized speech/behavior), and negative (anhedonia, blunted affect, apathy). According to DSM-5, a patient can be diagnosed with schizophrenia if he/she has two or more of the active symptoms listed above for at least one month, with one being a positive symptom, lasting for over six months. Our patient exhibited these symptoms and their onset must have been at least six months before the diagnosis of schizophrenia. Impairment in one of the major areas of functioning must also be present. This was displayed through our patient's declining school performance, self-care, and interpersonal relations. To help reduce her current symptoms, she was started on Abilify (aripiprazole) 30 mg PO daily (started initially and then discontinued after two weeks), LAIs Aristada (aripiprazole lauroxil) 1064 mg and Aristada Initio (Aripiprazole lauroxil) 675 mg IM, and Depakote (valproate) 500 mg. Abilify (aripiprazole) is an atypical antipsychotic that acts as a dopamine D2 receptor (D2) and serotonin 5-HT1A receptor (5HT1A) partial agonist, and serotonin 5-HT2A receptor (5HT2A) antagonist [10]. A second-generation antipsychotic, aripiprazole blocks dopamine and serotonin receptors but has fewer extrapyramidal side effects (acute dystonia, akathisia, parkinsonism). Aristada, containing aripiprazole lauroxil, is a long-acting injection of the atypical antipsychotic agent. Our patient received Aristada (aripiprazole lauroxil) 1064 and 675 mg for nearly two months. She was initially started on oral Abilify (aripiprazole) for two weeks before being given IM Aristada (aripiprazole lauroxil) to establish medication tolerance. Depakote (valproate) was added a few weeks post-IM medication due to ongoing psychosis and manic episodes. Depakote (valproate) is a drug used to treat mood episodes in bipolar disorder. Its mechanism of action is unknown, but it is believed to increase the level of gamma-aminobutyric acid (GABA) in the central nervous system (CNS) and inhibit voltage-gated Na channels [11]. These medications were added progressively. We continued to monitor the improvement in the patient and also track for side effects from antipsychotic/mood stabilizer medications such as extrapyramidal symptoms.

Our patient refused medication initially. As time progressed, she slowly agreed to take the medication and eventually stated that she believed she was feeling better. Although she continued to display signs of disorganized speech/behavior during weekly updates, it was evident that the patient was becoming more cognizant of her behavior, her talkativeness, and her delusion of being pregnant that she carried consistently throughout her stay at the center. Upon further analysis of the patient’s progression, it was determined that although she was initially evaluated and diagnosed with schizophrenia, her symptoms of mania such as excessive talkativeness and flight of ideas can also indicate a diagnosis of schizoaffective disorder. Schizoaffective disorder, although similar to schizophrenia, also includes mood disorder. According to DSM-5, schizoaffective disorder is defined as a major mood episode (bipolar type-mania or depressive type) along with all the criteria for schizophrenia. However, the symptoms of schizophrenia (hallucinations, delusion) should be present for ≥2 weeks without any major mood episode [12].

Substance abuse in patients with schizophrenia has been studied extensively. Studies have shown cannabis abuse as a stressor that induced a relapse of psychosis in patients with schizophrenia [13]. People at risk of schizophrenia are more vulnerable to substance abuse due to multiple factors ranging from genetics to changes in their mesolimbic brain activity involving the reward pathway. Substance abuse has been correlated to poor outcomes for patients through low treatment compliance. In a study by Arscnault et al. [13], regular cannabis use in the younger adolescent population had an increased risk of schizophrenia in adulthood. In a longitudinal study in New Zealand, participants aged 15 to 18 were assessed for regular cannabis use. Results portrayed that participants who utilized cannabis in that age group showcased greater symptoms of schizophrenia at age 26 compared to those who did not use it. Research based on 1055 participants showed that cannabis users had a 1.6 to 1.8 times greater chance of exhibiting psychotic symptoms [13]. This idea of substance use and its link to schizophrenia is vital in the case of our patient since she began to engage in tetrahydrocannabinol (THC) during her adolescence. Her previous and recent medical reports revealed urine analysis positive for THC. We are unsure how long the patient may have been using cannabis and in what quantity as these are important factors in understanding the relationship between the drug's impact and her mental health and recovery. 

Due to the circumstances of the patient's disappearance and the suspicious identity of the perpetrator from the initial police encounter, the patient was suspected of being a victim of human sex trafficking. In the following portion of the discussion, we will discuss pseudocyesis and the Stockholm syndrome in relation to the patient and how they can be probable factors to consider in terms of human trafficking. At the time of the patient's arrival at the center, she had had no contact with her family for four years and was reported missing in her home state. She was noted to be hypersexual, asking for male escorts to be delivered to the center and preoccupied with the notion of sex. She also kept mentioning how she was pregnant and insisted on repetitive pregnancy testing. This condition is known as pseudocyesis. Multiple studies have suggested that biological factors are important in pseudocyesis development. Neuroendocrine changes or disturbances have been recognized as factors that play a role in its development. Disturbances in the hypothalamic-pituitary-ovarian axis, although not necessarily unique to pseudocyesis, are commonly seen in its pathology [14]. Studies examining neuroendocrine factors in pseudocyesis have suggested that women displaying this condition might encounter enhanced nervous system activity or CNS impairment. In addition, a lack of dopamine is frequently observed in pseudocyesis. Schizophrenia contributes to notable dopamine hypo-activity in various regions of the brain. Dopamine in the CNS increases the possibility of some neurochemical relationship between schizophrenia and pseudocyesis. As per the etiology of our patient’s pseudocyesis, there are many theories as to its exact origin. It could be related to sexual trauma she might have experienced as a possible sex trafficking victim or due to her schizophrenia diagnosis. Regardless, the treatment remains the same, a pregnancy test and an ultrasound which would confirm absence of fetal heartbeat. The patient was ordered numerous pregnancy tests during her time at the center which were all negative and after two weeks of treatment with antipsychotic medications, she began to realize that she was having delusions that she was pregnant, perhaps indicating an improvement in her mental status. 

With continued analysis of the patient, it was probable that she was a human trafficking victim as mentioned above. Whenever the patient was asked about her past residence she refused to make eye contact, became anxious, and made an effort to dismiss the subject. She was more willing to talk about the matter in the third person where she then mentioned that there was drug paraphernalia in the house and that she was not allowed to leave the house. She stated that she couldn’t talk much about her living conditions for the past four years as she was “not allowed to and has boundaries.” These signs and symptoms may be indicative of Stockholm syndrome, which includes affection for captors and empathy for their causes and objectives combined with animosity towards law enforcement or authorities, which is considered a coping mechanism for a captive or abusive situation [15]. People with Stockholm syndrome tend to form a psychological bond with their captors. These individuals also tend to empathize, connect, and identify with them. There is still a question about why this syndrome evolves in some captives but not with others. One proposition implies that this condition might be an example of a survival mechanism that was passed down from our ancestors where the threat of capture or death by alternative groups was typical and forming bonds with the captors could augment their chances of survival [15]. A different theory suggests that the intense emotional atmosphere and psychological climate of captivity or abuse can guide individuals to cultivate feelings of compassion for their abuser, particularly if they are exposed to kindness with time [15]. Victims might fine-tune their emotions to line up with their abuser, believing that cooperation and support rather than opposition could improve their safety [15]. When not harmed, victims might feel a sense of gratitude and even perceive their abuser as being compassionate [15]. 

It is crucial to debunk the myth that associates schizophrenia with violence. The reality is that most individuals with schizophrenia are not violent, and the stereotype perpetuated by media sensationalism is both misleading and harmful. Research proposes that around 10% to 15% of patients with schizophrenia display violent behavior, meaning anywhere from 85% to 90% do not [4]. Individuals with schizophrenia living in the community experience victimization rates that are 65 to 130% higher than the general population [16]. Particularly, the rate of these individuals being victims of violence is 75 to 120% greater than the general public [16]. The principal determinants of this heightened risk include those with more acute symptoms and elevated levels of substance use, with the severity of symptoms being the most crucial factor [16]. The fact that people with schizophrenia are more likely to be victims of violence than perpetrators underscores the need for greater empathy and support. Their heightened vulnerability can be attributed to several factors, including cognitive impairment and social isolation [16]. Tackling these issues involves not only advancing mental health care and support systems but also aiming to lessen stigma and discrimination [16]. Generating accurate and compassionate portrayals of mental health conditions in the media and cultivating awareness within communities can help transform perceptions and better the lives of those affected. The findings mentioned above highlight a crucial challenge for policymakers and service providers regarding patients with schizophrenia who face an increased risk of being harmed in the community compared to the risk they pose others. To address this issue, there needs to be an improved collaboration between the mental health sector and the law enforcement agencies. Police officers should gain specialized training and education about mental illness in order to handle such interactions with sensitivity and understanding [16]. In addition, integrating community-based crisis assistance from trained mental health professionals can improve responses to individuals in mental health crises and better protect those who are vulnerable. Also, early detection of these individuals who are deemed vulnerable can help prevent further detrimental effects to the prognosis of their mental health status due to stressors and trauma, ultimately disrupting this vicious cycle.

## Conclusions

In this case report, we explored the relationship between substance abuse and schizophrenia, and the vulnerability of patients with schizophrenia to human trafficking. We also recognized and targeted solutions that can help prevent the progression of this vicious cycle. Previous research demonstrated that extensive cannabis use at a young age may activate an underlying genetic predisposition to schizophrenia. Patients with schizophrenia present with heightened vulnerability arising from factors such as cognitive impairment and social isolation. In this case report, with the patient presenting with pseudocyesis and Stockholm syndrome, it is vital to consider the possibility that she was a victim of human trafficking. The reality that individuals with schizophrenia are more frequently victims of violence, rather than perpetrators, emphasizes the urgent need for increased empathy and support. To address these challenges, it is crucial to not only enhance mental health care and support systems but also to combat stigma and discrimination by improving police officer training and education, integrating community-based crisis assistance to enhance response to patients with mental health crises, and increasing early detection of those who are vulnerable. Promoting more accurate and compassionate portrayals of mental health conditions in the media and fostering understanding within communities can help shift perceptions and improve the lives of those affected.
